# Engineering Efforts to Refine Compatibility and Duration of Aortic Valve Replacements: An Overview of Previous Expectations and New Promises

**DOI:** 10.3389/fcvm.2022.863136

**Published:** 2022-04-18

**Authors:** Stefano Rizzi, Sara Ragazzini, Maurizio Pesce

**Affiliations:** ^1^Tissue Engineering Unit, Centro Cardiologico Monzino, Istituto di ricovero e cura a carattere scientifico (IRCCS), Milan, Italy; ^2^Department of Chemistry, Materials and Chemical Engineering “Giulio Natta”, Politecnico di Milano, Milan, Italy

**Keywords:** calcific cardiac valve disease, valve substitution, tissue engineered heart valves, scaffold design, mechanical stress, TAVR, SAVR

## Abstract

The absence of pharmacological treatments to reduce or retard the progression of cardiac valve diseases makes replacement with artificial prostheses (mechanical or bio-prosthetic) essential. Given the increasing incidence of cardiac valve pathologies, there is always a more stringent need for valve replacements that offer enhanced performance and durability. Unfortunately, surgical valve replacement with mechanical or biological substitutes still leads to disadvantages over time. In fact, mechanical valves require a lifetime anticoagulation therapy that leads to a rise in thromboembolic complications, while biological valves are still manufactured with non-living tissue, consisting of aldehyde-treated xenograft material (e.g., bovine pericardium) whose integration into the host fails in the mid- to long-term due to unresolved issues regarding immune-compatibility. While various solutions to these shortcomings are currently under scrutiny, the possibility to implant fully biologically compatible valve replacements remains elusive, at least for large-scale deployment. In this regard, the failure in translation of most of the designed tissue engineered heart valves (TEHVs) to a viable clinical solution has played a major role. In this review, we present a comprehensive overview of the TEHVs developed until now, and critically analyze their strengths and limitations emerging from basic research and clinical trials. Starting from these aspects, we will also discuss strategies currently under investigation to produce valve replacements endowed with a true ability to self-repair, remodel and regenerate. We will discuss these new developments not only considering the scientific/technical framework inherent to the design of novel valve prostheses, but also economical and regulatory aspects, which may be crucial for the success of these novel designs.

## Heart Valve Pathology: Biological Causes and Current Solutions

### Calcific Disease of the Aortic Valve

Heart valve pathologies have been described as a cause of disability and death since the seventeenth century. They still remain today a relevant contributor of loss of physical comfort and reduction of longevity and result in a considerable socio-economic burden (1, 2). Diseases of the cardiac valves can be divided into two main categories, namely congenital pathologies (e.g., the malformation of the aortic and pulmonary valves, the Ebstein's Anomaly, the Fallot tetralogy or the bicuspid aortic valve), with an impact especially during the neonatal period and infancy, and acquired pathologies which, depending on the etiology, can have an impact at all ages (i.e., the rheumatic or the infectious heart valve disease) or in the elderly (e.g., calcification of the mitral and aortic valves) (1, 3, 4). In this framework, a major contribution to the increase in the overall impact of cardiac valves pathologies worldwide is the rapid increase of conditions leading to the aortic valve (AoV) stenosis, specifically “calcific aortic valve disease” (CAVD)-a disease correlated primarily to aging (1, 3, 5, 6) with an important sex-related component (7). We will refer below to prostheses to treat CAVD, considering that those employed to treat other pathologies are very similar in design and performance.

**Figure 1:**
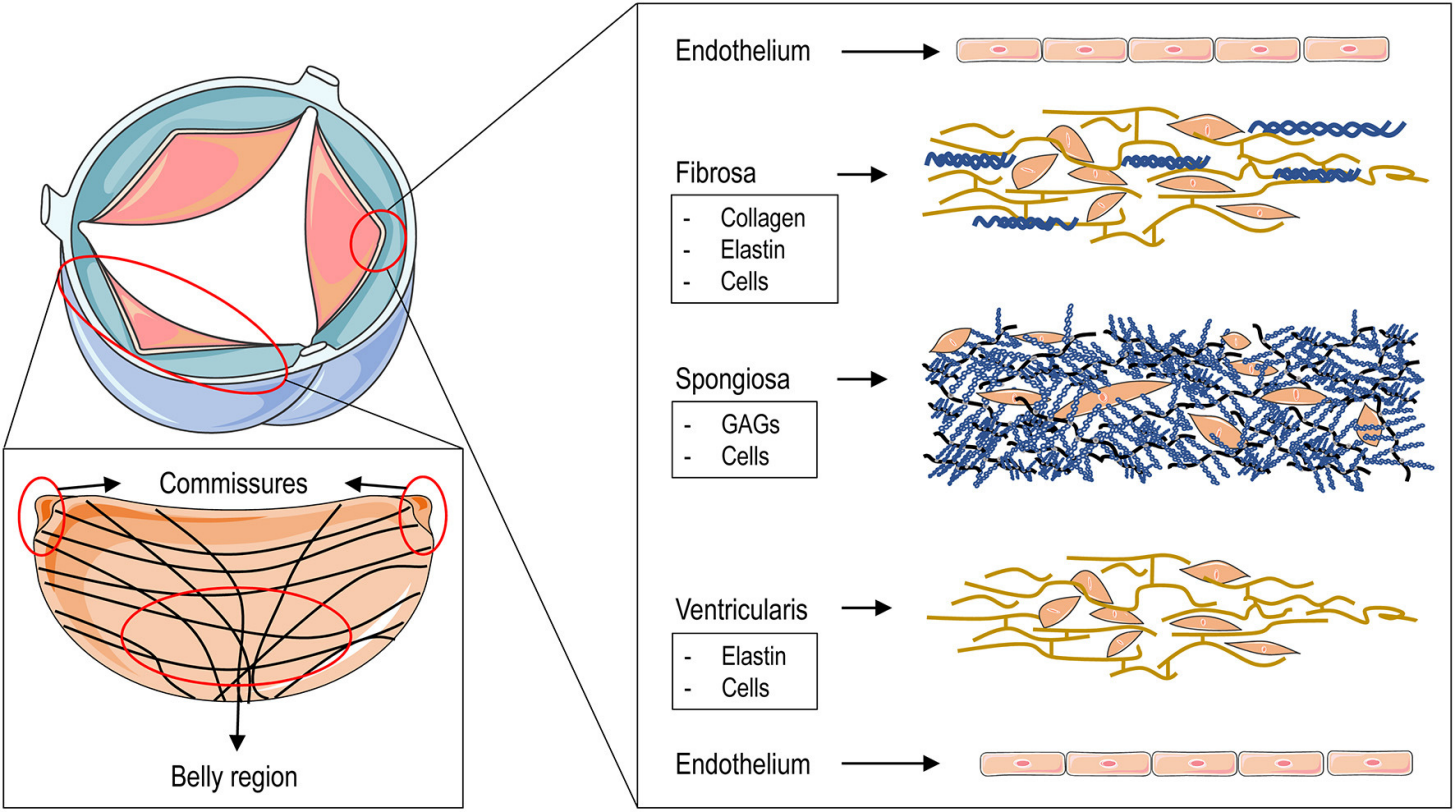
The figure illustrates the structure of the aortic valve and that of the different layers composing the three leaflets. The upper left drawing represents an aortic valve in the open position. The large area in red encircles a complete leaflet to show (in the lower left panel) the fine structure of the collagen fibers that are arranged circumferentially and which cross at the level of the “belly” region starting from the commissures. The small area encircling the tip of the cusp leads to an “exploded” view of the fine structure of the three leaflets layers with - ordered from the aortic side (top) to the ventricular side (bottom) - the fibrosa, the spongiosa and the ventricularis with their main “interstitial” cellular and matrix components. On the two sides of the leaflets a layer of valve endothelial cells is lined up to cover the basal membrane.

The AoV is composed of three leaflets, each of which comprises three laminas: the *fibrosa*, the *spongiosa* and the *ventricularis*, each with different structural and mechanical characteristics. The *fibrosa*, the layer associated with the outflow, or aortic, side of the leaflet, is predominantly composed of collagen fibers arranged circumferentially in parallel bundles and crossing with a typical “χ” geometry at the leaflet “belly” portion, surrounded by a matrix rich in elastin (8) (Figure 1). This is the layer that confers the maximal resistance of the leaflets to the compression forces acting on the aortic side, when the valve closes in diastole, and which can reach 80–120 mmHg (8). The *ventricularis*, the layer associated with the inflow side of the leaflet and facing the ventricular cavity, is a curvilinear structure mostly composed of elastin fibers oriented along the radial direction (Figure 1). The recoiling of these fibers supports the closure of the valve during the transition from systole (valve open) to diastole (valve closed) in the cardiac cycle (9). The *spongiosa*, finally, contains primarily glycosaminoglycans, a material with relatively low elastic modulus and an essential isotropic structure, which provide the deformability function of the valve leaflets and which serve to absorb the excessive mechanical forces (10). The three AoV layers are populated by specialized fibroblast-like cells, the so-called valve interstitial cells (VICs) (11). Although they are present in each of the valve layers, VICs are mostly abundant in the *spongiosa*, where they contribute to repair the abundant extracellular matrix continuously exposed to mechanical workload. VICs have heterogeneous phenotypes depending on the developmental and pathologic status of the valve (12).

The evolution of aortic valve pathology begins with the occurrence of micro-ruptures of the endothelial layer covering the leaflets, especially on the outflow surface of the valve, due to perturbations of the shear forces. As with the initial events of atherosclerosis, these ruptures cause lipid infiltration and recruitment of inflammatory cells (13–15). This, in turn, determines a release of inflammatory cytokines and an oxidative stress burst that lead to pathological activation of the resident VICs whose functions are altered under these conditions. Specifically, these cells participate directly in the inflammatory process by responding *via* Toll-like receptors 2/4 to inflammatory signaling and, in turn, secreting an array of inflammatory cytokines (16). VICs also undergo a modification in their phenotype from one of matrix-repairing to one of matrix accumulating/remodeling with the potential to cause thickening of the leaflets-the so-called valve “sclerotic” phase-which is considered the first pathologic event in valve disease (13). The phase of AoV sclerosis persists in a clinically silent fashion until the beginning of the more rapid phase of valve calcification, characterized by transformation of VICs into calcific cells. These “osteoblastic” VICs have the ability to secrete initially small, but subsequently larger, calcific nodules that progressively deform the leaflet structure (17). This causes variations in the motion of the leaflets, incomplete valve closure at diastole and regurgitation with ensuing compromisation of heart function. While inflammatory signaling is generally connected to the initial VICs matrix remodeling activity in the sclerotic phase, transformation from “activated” to osteoblastic “VICs” has been also linked to mechanical factors. In this respect, it has been hypothesized that the progressive hardening of the matrix surrounding VICs due to their remodeling activity prompts the activation of mechanosensitive-dependent pathways setting progression of the cells toward a calcific phenotype (18, 19).

### Clinical Options for Valve Replacement

Until the beginning of the current century, surgical aortic valve replacement (SAVR) has been considered the elective option for surgical treatment of heart valve pathologies. Although highly effective, this is an invasive procedure requiring temporary cardiac arrest and extracorporeal circulation, which exposes patients to complications and side effects (20). For the majority of patients, the choice of the replacement device for SAVR is either a mechanical or a bioprosthetic valve. In a minority of patient's other options are adopted. These include the recuspidalization with autologous pericardium (the so-called Ozaki procedure) or transposition of the autologous pulmonary valve into the aortic position with replacement of the pulmonary valve with an aortic homograft-the Ross procedure adopted commonly for infants and children with congenital valve defects/stenosis and young adults (21–25).

A recent novel possibility to restore the functionality of a diseased aortic valve with reduced peri-procedural side effects involves trans-catheter aortic valve replacement (TAVR). This technology, exploiting the pliability of pericardial tissue, allows deployment of a completely functional prosthesis using a minimally invasive procedure. These valves are currently approved for patients with severe, symptomatic aortic stenosis in all surgical risk categories given the favorable outcomes in the postoperative period (26, 27). Despite that TAVRs are based on a novel design and can be implanted with enormously lower risks, they still carry severe problems related to structural deterioration analogous to that of bioprosthetic grafts. For this reason, they are rarely implanted in patients younger than 60–65 years of age in accordance with the guidelines (28).

## Polymeric vs. Tissue-Engineered Valve Replacements

Given the shortcomings of contemporary valve replacements, over the years, innovative designs have been sought using different approaches and manufacturing philosophies with the aim, in certain cases, to maximize the ease and minimize the cost of the approach (i.e., polymeric valves, PVs), and in other cases to ensure the maximal biocompatibility (tissue engineered heart valves, TEHVs). We will discuss these two approaches separately, highlighting advantages and drawbacks.

### Polymeric Valves

Polymeric valves are manufactured with elastomeric polymers by a simple fabrication procedure using molds. Typically, the process involves “injection molding” whereby synthetic (e.g., polyurethane or polystyrene) (29, 30) or natural polymers (e.g., fibrin) (31) are injected into tri-leaflet molds that give rise to complete sutureless valves, and can be readily mounted onto posts for implantation. This design provides advantages including easy scalability, low cost, natural hemodynamic performance and a relatively high long-term durability comparable to that of mechanical prostheses (32). On the other hand, when employed in animal valve replacement studies, polymeric valves, at least initially, did not lead to encouraging results due to calcification, thrombus and fibrous capsule formation, resulting in implant failure (33–35). Despite these shortcomings, in 2010, Quintessenza et al. (36) published a clinical study performed on 126 patients surgically treated with bicuspid pulmonary polytetrafluoroethylene (PTFE) valves. In particular, two types of valves with different thicknesses were used; the first was made with porous 0.6 mm PTFE while the second with non-porous 0.1 mm PTFE. Six patients treated with the porous PTFE valves needed reoperation due to leaflet calcification. In contrast, non-porous valves were less prone to stenosis as the lack of porosity prevented cellular in-growth and thickening (37). Moreover, 0.1 mm PTFE valves were characterized by higher leaflet mobility and lower transvalvular gradients (36). Stasiak et al. (29) recently introduced the so-called Poli-Valve (38)-a styrene triblock copolymer valve obtained by injection molding. This technique, besides being inexpensive and highly reproducible, appears to allow an optimal anisotropic distribution of forces on the leaflets and the polymeric fibers (39, 40) resulting in maximal mechanical durability due to a similar collagen fiber orientation to that of native heart valve tissue (39). The valve was bench-tested and validated according to ISO standards. Moreover, preliminary *ex vivo* and short-term *in vivo* feasibility tests were done, showing a good biocompatibility in the absence of mechanical failure and regurgitation. The lack of long-term tests *in vivo* still raises the question as to whether these valves offer an advantage over the most advanced mechanical replacements, in particular concerning the need for anti-coagulation therapies to prevent formation of thrombi on the surface of the leaflets.

### First Generation TEHVs: Advantages and Drawbacks

The general strategy to derive living replacements resembling native tissues was introduced in 1993 by Langer and Vacanti (41). These Authors, in their initial proposition of tissue engineering, defined three essential steps consisting of, (*i)* to seed autologous or allogenic-compatible cells inside scaffolds pre-fabricated with biocompatible/biodegradable materials, (*ii)* to enable tissue formation in bioreactors by exposing the tissue constructs to controlled mechanical loading and, (*iii)* to promote final tissue maturation, exploiting the ability of the pre-seeded cells to interact with circulating cells to complete the final evolution of the tissue constructs toward native leaflets (41). If induced to mature with appropriate instructing stimuli, the tissue constructs could therefore have regeneration and growing capacity. In the valve pathology scenario, this ability to grow would be especially indicated for use in pediatric patients, for whom the possible failure of the implants is compounded by the inability of the new valve to grow with the individual, making continuous surgical procedures necessary (42).

#### *In vitro* Strategy

**Figure 2:**
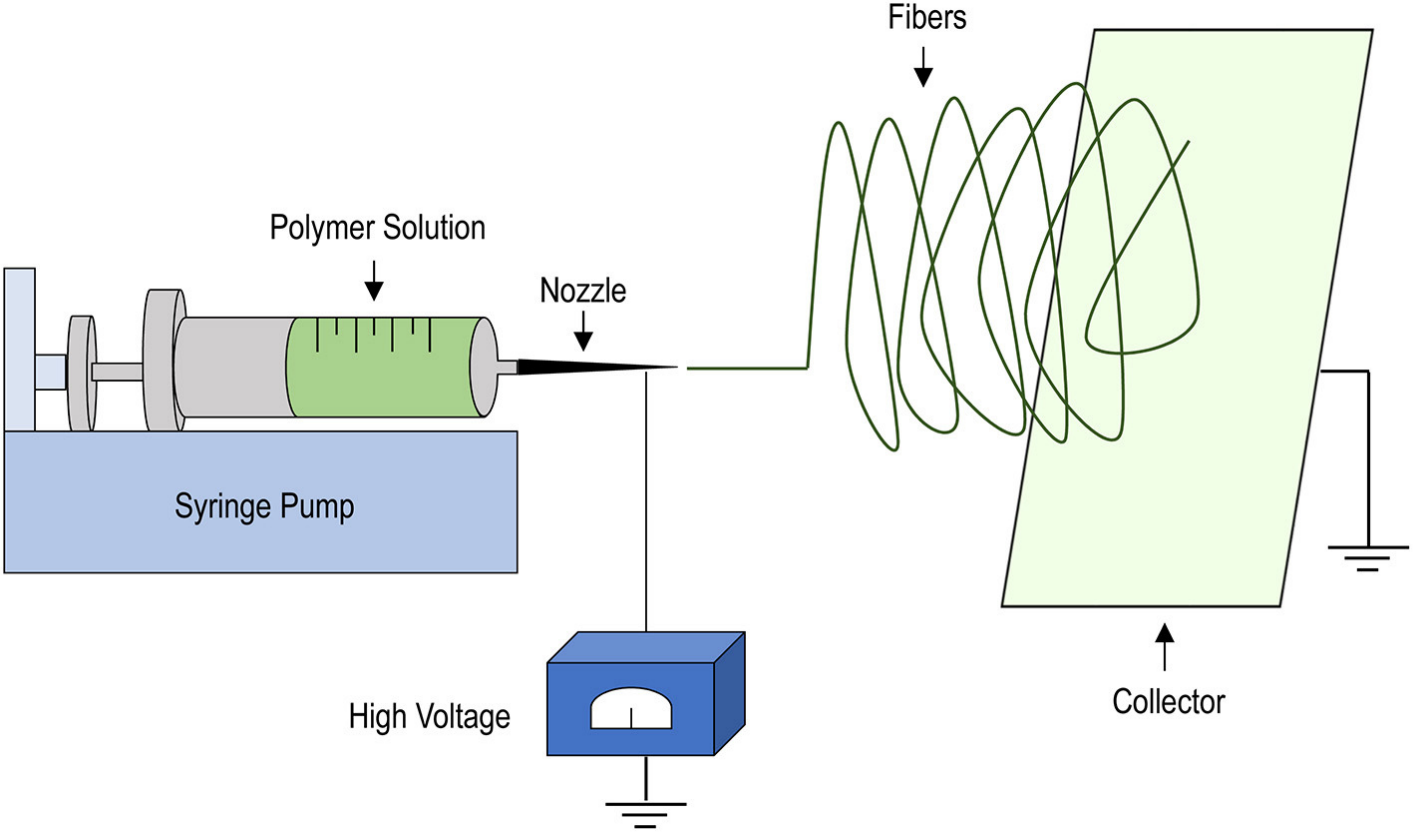
Schematics of the electrospinning procedure. A liquid polymer solution is loaded into a syringe mounted into a syringe-pump and set to flow at defined rates through a nozzle of variable diameters (depending on the operational needs). The application of strong electric field allows the polymer to deposit onto a collector, consisting of a rotating mandrel or, as shown, a rotating plate onto which a non-woven scaffold can be manufactured due to solvent evaporation and solidification of the polymer fibers. Scaffold properties, such as porosity, fiber dimension and thickness can be easily adjusted by varying the dimension of the nozzle, the extrusion speed, the intensity of the electric field and the rotation speed of the collector.

During the years, several TEHVs manufactured with the classical tissue engineering approach [that we cite here as the “*in vitro strategy*” (32)] have been developed using various materials and manufacturing procedures. Particularly important in the scenario of this first type of valve replacement is the polymer deposition, or electrospinning technique (Figure 2). Electrospinning allows deposition of polymeric fibers by exploiting the ability of electric fields to direct these fibers onto rotating mandrels or planar/curvilinear surfaces (43). Given that the manufacturing process is performed by extruding liquid polymers through nozzles with different diameters, the dimension of the fibers, and the porosity of the scaffolds can be easily controlled. Moreover, by adjusting the rotation speed and/or the motion of the spotting surfaces, the orientation of the fiber deposition can be adjusted, thus allowing one to mimic, to a certain extent, the mechanical properties of the natural leaflets, thus allowing the scaffold to offer resistance to mechanical forces inherent to valve motion (44–46).

A first remarkable biodegradable polymeric material that was tested to produce electrospun meshes for assembly of valve scaffolds was polyglicolic acid (PGA). For example, in 1995, Shinoka et al. (47) manufactured a single leaflet valve constructed from non-woven PGA mesh sheets. The synthetic valve was seeded with ovine fibroblasts and endothelial cells, and then cultured up to 10 weeks. Initial *in vivo* tests performed on *in vitro* cell-seeded PGA scaffolds in lambs indicated absence of stenosis, especially when cells used to populate the scaffolds where autologous. In a more recent study, published in 2011, Schmidt et al. (48) tested a tri-leaflets synthetic pulmonary valve implanted in sheep using minimally-invasive surgery. The valve was fabricated starting from nonwoven PGA meshes coated with poly-4-hydroxybutyrate (P4HB) using a heat-application welding technique. Scaffolds were then cultured *in vitro* with autologous myofibroblast and endothelial cells using dynamic bioreactors. *In vivo* tests confirmed adequate tissue formation and proper opening and closing of the valve.

In subsequent years, combinations of other polymers have been assessed to fabricate functional TEHVs. One example is the valve developed by Gottilieb et al. composed by a mixture of PGA and poly-L-lactic acid (PLLA) fibers. The valve was assembled commencing with non-woven sheets containing PGA and PLLA fibers in a 1:1 ratio bonded by manual and machine needle punching. The scaffold was subsequently seeded with ovine bone marrow cells and cultured for 4 weeks prior to *in vivo* implantation (49). Valve insufficiency was, however, noted after 6 weeks. Moreover, although valve conduit diameter remained stable up to 20 weeks post-implantation, increasing valve regurgitation, corresponding to decreasing cusp length over time, was observed. The application of Hasan et al. (50) of a blend of polycaprolactone (PCL) and PLLA is of particular interest as it combined the high stiffness and the mechanical properties of PCL with the cell adhesive properties of PLLA, with cell-spreading and metabolic activity providing encouraging results.

The general and major shortcoming in the use of bioabsorbable materials such as PCL, PLLA or PGA is the failure to maintain a constant leaflet geometry and mechanical coherence, which results in retraction and thickening of the leaflets and valve insufficiency and regurgitation (49, 50). One of the most striking examples of this effect was described by Hoerstrup et al. (51) with respect to a valve composed by electrospun sheets of PGA coated with P4HB using a welding technique. Even if adapted for minimally invasive procedures, the scaffold design was insufficient to maintain a mechanical coherence over time after *in vivo* implantation, with general deterioration of the geometry and an overall thickening of the leaflets (48) which caused regurgitation and insufficiency (48).

The “retraction” and “compaction” effects of TEHVs manufactured with biodegradable materials are mainly cell-mediated, and derive from the uncontrollable matrix degrading activity of the pre-seeded cells, or the cells recruited from the circulation (mainly monocytes/macrophages). While cell-mediated degradation can be reduced by optimizing the mechanical and surface characteristics of the polymers (e.g., stiffness, rigidity, wettability) and/or by performing functionalization of the electrospun fibers with natural or synthetic materials, an important factor is also the influence of mechanical load transmission from the scaffold to the cells. In fact, the unequal distribution of the strain forces on the curvilinear structure of the aortic valve leaflets, i.e., from the belly of the leaflet to the commissures (8), creates zones where the leaflet experiences maximal compression forces and other zones where forces are significant lower. Given the general mechano-sensitivity of adhering cells and, more in particular, that of the cells generally employed in valve tissue engineering (e.g., mesenchymal cells, valve cells, and fibroblasts) (19, 52) and the propensity of these cells toward a matrix remodeling and “pulling” phenotype when subjected to mechanical stress (53, 54), an essential component in TEHVs design is the possibility to achieve a mechanical adaptation of the cells to the microenvironment. According to findings by Cox et al. (55) this condition may be achieved, at least in part, by exposing TEHVs constructs to controlled mechanical stimulation, which may promote maturation of the tissue with a native distribution of collagen fibers and a lower propensity to remodel over time after implantation. The combination of natural with synthetic polymers could, finally, prevent a precocious onset of the maladaptive cellular responses observed in TEHVs manufactured with bio-absorbable non-woven materials, thus enabling the possibility to obtain structures with more stable and constant mechanical properties (56). However, the lack of long-term studies (49) and of an exhaustive knowledge of the cell-material interaction, nowadays excludes the transfer of these engineered valves to clinical practice.

#### *In-situ* Strategy

The second approach for generating TEHVs is the so-called “*in situ* strategy,” which exploits the ability of the human body to promote new tissue formation starting from an acellular implant due to the recruitment of circulating cells. In this setting, the postoperative adhesion of autologous cells to the scaffolds is a crucial event expected to provide a structure with performances as similar as possible to that of the native valves (57–59). An example of this approach is the electrospun valve fabricated by Kluin et al. (60) using a novel supramolecular elastomer; bis-urea-modified poly-carbonate (PC-BU). The function of the valve manufactured under these conditions was studied *in vitro*, while cellular recruitment and new tissue formation were evaluated during a long-term follow-up (12 months) in ovine model. Both phases of the study produced satisfactory results. In fact, the valves exhibited good functionality in terms of leaflets mobility, and did not show major signs of stenosis and thrombus and maintained a stable geometry and good cellular colonization *in vivo*.

Another example of an *in situ* TEHV is that recently developed by Coyan et al. (61). The scaffold was fabricated using a double component deposition (DCD) electrospinning strategy employing poly- (ester carbonate urethane)-urea (PCUU) as a material. With this procedure, the authors were able to obtain valves with a broad range of geometries suitable for stentless, stented and transcatheter applications (62). The valves were evaluated 24 h post implantation in a porcine model. Immediate postoperative analyses showed good valve kinematics and, at explant, no sign of stenosis, structural deterioration or thrombus was observed, even if the absence of a long-term study prevented full assessment of the effective regenerative potential of this PCUU valve.

An interesting *in vivo* study published by Emmert et al. (63) exploited a valve that combined both *in vitro* and *in situ* approaches. Briefly, a tri-leaflets heart valve was fabricated commencing with non-woven PGA sheets coated with P4HB as previously described (51). The scaffold was then seeded with ovine vascular derived cells and cultured in a dynamic bioreactor for 4 weeks. Before being implanted, the valve was decellularized to obtain a structure suitable, as much as possible, for re-colonization by autologous cells (64). At the end of the follow-up period (12 months), the valve exhibited good performance and tissue remodeling comparable to that of the native aortic valve.

Currently, concordance is lacking on the best approach to be followed given the pros and the cons of the two approaches. In fact, while the *in vitro* approach seems more appropriate to keep the phenotype of the cells under control until tissue maturation is complete, it requires complicate and time-consuming tissue engineering procedures which need to be performed in compliance with the rules for good manufacturing practice (GMP), and additionally necessitates huge monetary investments inappropriate for the increasing demand. In contrast, *in situ* TEHVs, which lack a living component, could be produced with an off-the shelf strategy at enormously lower costs and could be easily implemented into the market. The shortcoming of this approach is that the efficiency of the *in situ* recellularization and tissue maturation is less controllable, given the anticipated patient-to-patient variability due to the effects of age, risk conditions and pathological settings, which could lead to a variable degree of inflammation and failure (65).

### Second Generation of TEHVs: New Materials and Designs

In view of the growing awareness of the maladaptive interactions between cells and scaffolds used to produce TEHVs, more complex manufacturing concepts are now emerging based on more systematic views of the cells/scaffolds interactions (66) and the recognition of the role of the forces dominating cellular mechanosensitivity of the cells (20, 67). Central to this second-generation design is the change from scaffolds made of randomly interleaved fibers, to a design that is more compliant with the distribution of the strain and compression forces acting in the kinematics and mechanical loading of the natural valves. The idea underlying this new concept derives from the evidence that the natural ECM fibers in the valve are deposed from the very beginning of valve development mainly with anisotropic patterns instructed by mechanical forces, and that the cells residing within the anisotropically deposited fibers are adapted to maintain a quiescent phenotype (Figure 1) (68). In order to achieve this aim, one of the current trends is to manufacture scaffolds with oriented deposition of ECM components (e.g., collagen) by mechanically forcing cells to deposit fibers according to defined geometric patterns, and/or to employ polymers that can be deposited with anisotropic patterns in 3D. A further step in this biomimicry approach is the attempt to implement the natural tri-layered valve structure in scaffold design. An example of this new design has been provided by Masoumi et al. where a three-layer scaffold included an anisotropic fibrous layer deposited between two coatings of electrospun fibers. Cells were then seeded in an attempt to obtain a fully engineered heart valve with layers resembling the native structure of the aortic valve tissue (69). Despite the fact that the resulting valve differed from the native valve in its organization of the ECM (for example, that an anisotropic layer of PGS represented the highly isotropic *spongiosa* layer), this type of scaffold gave good results when mechanical performance and maintenance of cellular viability was considered (70). Unfortunately, the lack of *in vivo* translation of this valve to date does not allow inferences on its behavior in a living organism. In a second example, Eslami et al. (71) employed a hydrogel made with a mixture of methacrylated hyaluronic acid and methacrylated gelatin, into which mitral valve interstitial cells were incorporated followed by its incorporation into a PGS-PCL electrospun scaffold. The author's speculated that this approach produces a more favorable environment for the remodeling of the ECM by the cells after *in vivo* implantation of the valve. Indeed, encasing cells into hydrogels before seeding a scaffold might mitigate the matrix digestion/remodeling activity of the cells and at the same time would favor the *de novo* deposition of matrix without affecting the mechanical function of the PGS-PCL layer. This may be particularly interesting considering that the behavior of the cells (and the resultant activation status) can be potentially modulated by mechanical tuning of the hydrogels characteristics, thereby crucially contributing to maintain them in a quiescent/self-renewing phenotype.

Numerous efforts toward the production of valve scaffolds with anisotropic mechanical characteristics have been made using novel fabrication techniques. For example, in a recent study by Wunner et al. (72) a polymeric scaffold with a highly controlled microarchitecture was manufactured using an “electrowriting” technique, which involves high voltage guided printing of a solvent-free, melted polymer onto a laterally sliding aluminum collector. Using this approach, the authors were able to orientate the polymeric fibers (made with medical grade PCL) to mimic that of the collagen and elastin fibers of the natural valve, resulting in a mechanical behavior comparable to that of native valve leaflet (73). Another method that has been exploited with the same aim has been developed by Moreira et al. (74) who introduced textile reinforcements into a scaffold containing a thin valve electrospun layer and fibrin cell-laden gel to confer anisotropic resistance against the forces acting in the valve motion cycle, which resembled the arrangement of collagen bundles of the native *fibrosa* layer. Preliminary bench testing of the resulting valve after 21 days dynamic conditioning, proved that mechanical stimulation enhanced matrix deposition (in particular collagen) by the cells, thus showing the versatility of a “mixed” fabrication approach to elaborate a design that more closely resembled the natural valve architecture. The utility of a tailored deposition of valve scaffolds fibers is, however, still under question, especially from the perspective of long-term scaffold remodeling after *in vivo* implantation. In fact, for example, Uiterwijk et al. (75) showed that the orientation of the collagen fibers in *in situ* TEHVs that were manufactured with isotropic or anisotropic fibers deposition and implanted for 1 year in sheep did not resemble the original arrangement of the fibers in the scaffold, but was instead dictated by the prevailing mechanical forces after implantation. While the authors concluded that the fiber's anisotropic deposition is insufficient to dictate the way the scaffold-populating cells mechanically adapt and deposit new matrix components, it has been discussed that other factors, such as the relatively rapid degradability of the scaffold and inflammatory response, may contribute to override any instructive signals provided by the original geometry of the implant (76).

Finally, a promising technology that is still in its infancy but will undoubtedly provide a decisive future impact in cardiovascular medicine, is that of three-dimensional (3D) printing of valve scaffolds or direct bioprinting of valve leaflets using cell-laden polymers (77). The advantage of this manufacturing technology is that 3D printing/bioprinting allows deposition of matrix components with precise patterning and also exploits a layer-by-layer positioning of materials and cells with a high control of the output geometry (with μm accuracy). Potentially, given the possibility to translate the actual geometry of valves via imaging system data (i.e., CT-scan), this technique could be used for personalized manufacturing of valves tailored to the individual patient with maximal hemodynamic performance and adaptability (78). Despite these advantages, 3D printable materials or, in particular, bio-printable hydrogel/cellular mixtures still suffer from the lack of mechanical strength (79–81), especially with respect to the need to withstand an intense mechanical workload. In keeping with this conclusion, for example, the collagen Type I bioprinted valve described by Lee and co-authors (77) was efficiently cellularized with human endothelial cells, but its mechanical characteristics were insufficient to meet the standards required for mitral and aortic valves *in vivo*. While other valve-specific printable polymers are currently undergoing investigation, including methacrylated hydrogels, such as gelatin and hyaluronic acids (82), and PEG-DA hydrogels (83), further work is necessary to improve the mechanical characteristics of 3D/bioprinted valves to produce realistic alternatives.

## Recellularization of Decellularized Natural Tissues

Considering the above highlighted shortcomings of fully engineered TEHVs resulting from the combination of cells with artificial scaffolds, a further strategy that still has appealing features for engineering living valves, is to introduce human cells *de novo* into decellularized animal-derived materials, such as entire valves or pericardium. Prompted by remarkable examples such as the re-engineering of decellularized whole hearts (84), this strategy appears a realistic alternative, especially when considering the possibility to engineer tissues with full regeneration capacity and maximal biocompatibility for use in pediatric and young patients. The advantage of tissue decellularization includes the possibility of employing animal scaffolds already endowed with mechanical characteristic that closely resemble that of diseased tissues. For heart valve engineering, for example, porcine valves and porcine/bovine pericardium are elective materials either for the maximal mechanical compatibility (especially with respect to valves) and the easiness of valve manufacturability (especially for pericardium). Before being employed in a tissue engineered construct, the decellularized materials need to meet specific requirements to ensure maximum immunological compatibility once implanted into the human body. In this respect, a principal element that needs to be addressed is the problem of xenoantigens, which is an overarching problem in the use of bioprosthetic valves as it is in vascularized solid organ xenotransplantation. Chemical cross-linking (using glutaraldehyde), which is normally performed to prepare bioprosthetic tissue for use in valve replacement procedures, fails to fully quench the immunogenicity thereof, including DNA and other cellular xenoantigens, such as Alpha-Gal (galactose-α-1,3-galactose) which, together with reactive aldehyde residues themselves, eventually lead to progressive deterioration of the valve (85, 86). In order to avoid the presence of xenoantigens, one possibility includes decellularization using ionic and/or non-ionic detergents (87, 88). Another possibility involves the use of “humanized” valve tissue through the generation of genetically engineered donor animals (89). Indeed, since the majority of human antibodies against the porcine material bind to the αGal epitope (90), the use of pigs with a knockdown in the GGTA-1 locus (GTKO) would not give rise to xenograft rejections due to the αGal epitope (91). Genetic ablation of other antigens, such as that encoding the SDa blood group or the N-glycolyl neuraminic acid offer potential advantages (91–94). The problem of residual immunogenicity of decellularized tissues is, however, not entirely resolved. In fact, there is a residual possibility of long-term rejection of decellularized tissues due to the persistence of residual contaminants deriving from the detergents employed, presence of ECM components that are not removed by the decellularization procedure and permanence of cellular debris not completely removed by post-decellularization washing procedures (95). This shortcoming might be overcome by elaborating a quality control system and toxicologic assessment of the decellularized material in view of clinical translation.

A second element to be considered in the employment of decellularized materials is the strategy to re-introduce the cells inside such scaffolds. In this respect, several unsuccessful attempts in the past have been performed based on culturing cells on the surface of the decellularized matrices (96, 97). The drawback of this approach is that decellularized matrices are mostly impervious to invasion by cells from the surface due to low porosity and permeability thereof. In this context our group developed a decellularization method for pericardium based on the employment of ionic and non-ionic detergents that both maintained the structural integrity and mechanical resistance of human (87) and porcine (98) pericardium and abolishes xenoantigens in the latter. Interestingly, when valve cells were statically cultured on the surface of the decellularized porcine pericardium, only minimal penetration was observed (98). In contrast, the increase in tissue permeability following decellularization rendered the tissue perfusable with an oscillating perfusion bioreactor (88), which led to a homogeneous distribution of the cells throughout the entire depth of the construct thereby effecting valve-like tissue maturation (99). Despite the lack, as yet, of confirmation of total biological compatibility of the recellularized tissue or the scalability of the method, the approach seems amenable to generate a fully engineered, personally tailored (for example, using recipient mesenchymal cells), living valve for selected classes of patients (e.g., infants/children and young adults).

## Conclusion

The present review describes past and present approaches in conceiving TEHVs and summarizes the current drawbacks that need to be overcome. While more historical design of TEHVs failed to consider the morphological design of matrices, which resulted in leaflets retraction/compaction and, thus, eventual failure, the overall trend emerging from more recent studies of new designs serves to elucidate the strict correlation between maintenance of proper cellular mechanosensitivity and the correct mechanical adaptation/maturation of the engineered tissues *in vivo*. This last consideration also underlines the urgent need of a more integrated work between engineers and biologists to come up with a systematic design of scaffolds and fine-tuning of material characteristics to minimize the cell-mediated effects in scaffolds remodeling. In the context of modern valve tissue engineering, decellularized matrices still appear promising for TEHVs fabrication, despite the potentially high manufacturing costs which may limit their availability and use in well-suited recipient classes.

## Author Contributions

SRi and SRa drafted the manuscript and the figures. MP revised the work and elaborated the final version of the article. All authors contributed to the article and approved the submitted version.

## Funding

MP is recipient of Institutional funding (Ricerca Corrente e Ricerca 5‰), both from Ministero della Salute, Italy.

## Conflict of Interest

The authors declare that the research was conducted in the absence of any commercial or financial relationships that could be construed as a potential conflict of interest.

## Publisher's Note

All claims expressed in this article are solely those of the authors and do not necessarily represent those of their affiliated organizations, or those of the publisher, the editors and the reviewers. Any product that may be evaluated in this article, or claim that may be made by its manufacturer, is not guaranteed or endorsed by the publisher.
